# Hip dislocation in a 3-year old girl after minor trauma: A rare case report

**DOI:** 10.1016/j.tcr.2025.101223

**Published:** 2025-07-09

**Authors:** Mirza Sivro, Đemil Omerović

**Affiliations:** a Department of Orthopedics and Traumatology, Cantonal Hospital Zenica, Zenica, Bosnia and Herzegovina; b Clinic of Orthopedics and Traumatology, Clinical Center, University of Sarajevo, Sarajevo, Bosnia and Herzegovina

**Keywords:** Hip injuries, Joint dislocations, Pediatrics, Plaster casts

## Abstract

Traumatic hip dislocations in children are rare injuries. They are mostly sustained after low-energy trauma in younger children, and after high-energy trauma in older children and adolescents. In 90 % of cases dislocation is posterior. Since this condition is an orthopedic emergency, it requires urgent reduction in order to avoid complications such as recurrent or habitual dislocation or avascular necrosis (AVN) of the femoral head.

A 3-year old girl presented sustained posterior dislocation of the right hip after twisting injury. Clinically, the right leg was shortened, and in the position of adduction, with limited passive motion of the right hip joint. Plain pelvis X-ray revealed right hip joint dislocation. Emergency closed reduction was made after sedation by Allis maneuver, and one and a half hip spica cast was applied. After three weeks, the cast was removed and patients referred to physical therapy with allowed weight-bearing on the injured leg. At 8-month follow-up, clinical and radiographic findings were normal.

Careful evaluation, complete history, thorough clinical exam and imaging are required in rare cases of pediatric traumatic hip joint dislocation, with prompt closed reduction, in order to achieve favorable clinical outcomes.

## Introduction

Hip dislocations of traumatic origin in the pediatric population account around 5 % of all dislocations [1]. They are mostly sustained after low-energy trauma in younger children, and after high-energy trauma in older children and adolescents [2]. Classification, which is mainly based on position of the femoral head in relation to the pelvis, divides these dislocations into four major types: posterior, antero-superior, antero-inferior, infracotyloid, with posterior dislocations being the most frequent one (in 90 % of cases). Clinically the child has pain, which is often projected to the knee, and refuses to walk or has the inability to ambulate. Diagnosis is most often made by clinical examination, plain pelvis radiography, and in case of fracture fragments and soft-tissue interposition, with CT or MRI [3]. Since traumatic pediatric hip dislocation is an orthopedic emergency, it requires urgent reduction in order to avoid complications such as recurrent or habitual dislocation or avascular necrosis (AVN) of the femoral head [4,5].

This case report presents the traumatic posterior hip dislocation in a 3-year girl after low-energy trauma, which was treated non-operatively after a successful reduction, and emphasises the importance of careful evaluation and prompt management of this rare injury.

## Case presentation

**Fig. 1:**
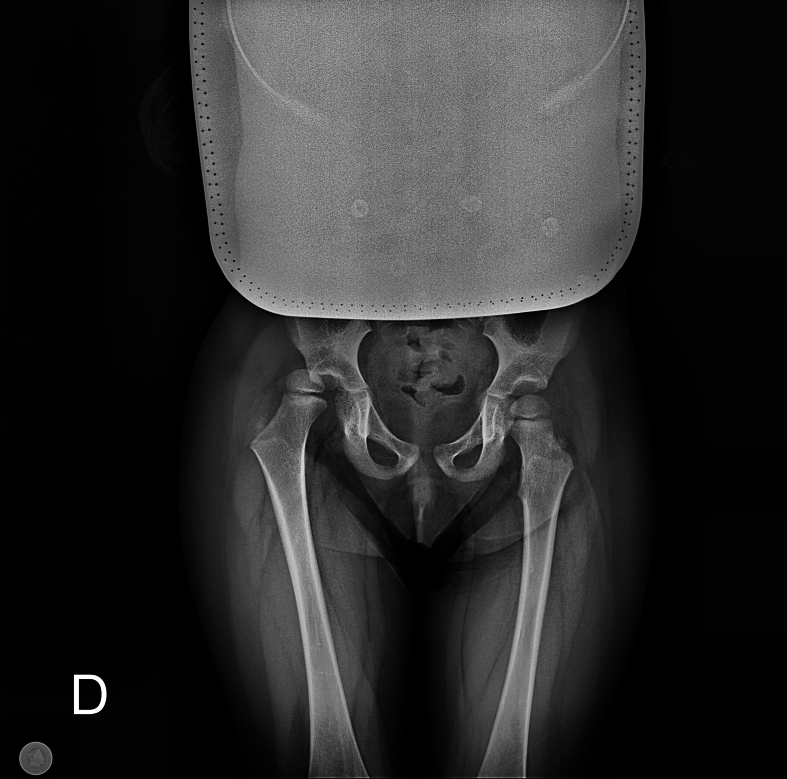
Pelvis X-ray at admission.

**Fig. 2:**
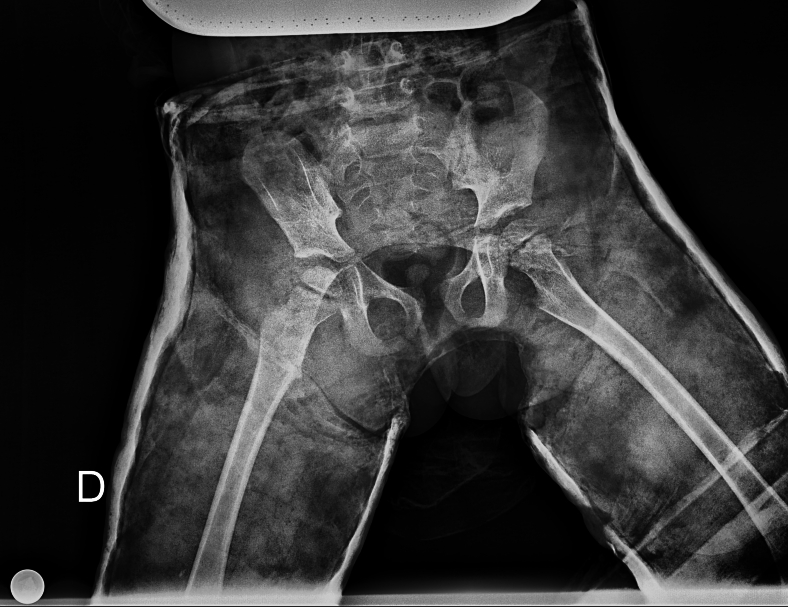
X-ray after reduction and cast application.

**Fig. 3:**
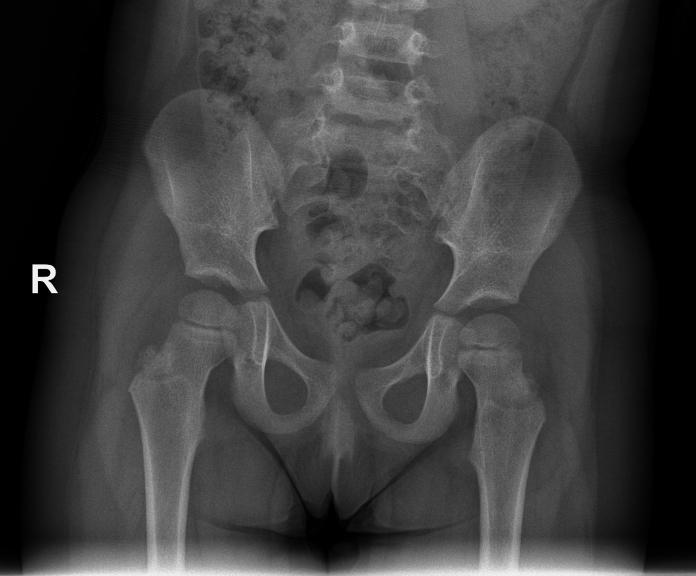
Pelvis X-ray at 8-month follow-up.

A 3-year old girl with her parents was referred to the orthopedic surgeon by the emergency department (ED) after a right hip injury. According to her parents, the girl was playing after she made a torsional motion of her right leg and then fell on the ground. Since then, she can't bear weight on her right leg. There was no history of earlier developmental dysplasia of the hip (DDH), nor any other medical condition, and family history for musculoskeletal diseases was negative. Clinically, the right leg was shortened, and in the position of adduction, with limited passive motion of the right hip joint. Plain pelvis X-ray revealed right hip joint dislocation (Fig. 1). Emergency closed reduction was made after sedation by Allis maneuver, and one and a half hip spica cast was applied (Fig. 2). Circulatory and neurological status of the right leg before and after the reduction were intact. Hip spica cast was maintained for three weeks, after it was removed, and the patient was referred to physical therapy and allowed to bear weight. At 8-month follow-up, active and passive motion in right hip, gait as well as pelvis X-ray (Fig. 3) were normal.

## Discussion

We reported on a rare case of traumatic hip joint dislocation in pediatric patients. The study of Avadi K et al. highlights the rarity of this injury, in which only 15 cases were reported in a single large trauma center during a 20-year period [2]. The low-energy mechanism of this injury was present in children under six years of age according to the same authors. At clinical examination, patients often complain about knee pain, and it is mandatory to exclude all other conditions associated with hip pathology in pediatric patients with hip and knee pain according to the age and clinical exam, which under differential diagnosis include: transient coxitis, septic arthritis, slipped capital femoral epiphysis (SCFE) and Perthes disease [6]. Careful evaluation of plain radiography prior to reduction and after reduction is necessary to rule out joint space asymmetry which may be due to incomplete joint reduction, especially in cases of spontaneous reduction and interposed soft tissue. This condition seeks additional diagnostic imaging, including CT and MRI. The most common complication includes osteonecrosis of the femoral head, which is related to time from injury to reduction and severity of the injury [7]. Although type of management after the reduction is not associated with the rate of AVN, further investigation, case series or larger studies are required to define postreduction treatment protocol, since there is no consensus established [8]. At our institution, simple and isolated dislocations of the hip joint in children are treated with closed reduction and 3-week immobilisation in hip spica cast after weight bearing is allowed.

## Conclusion

Careful evaluation, complete history, clinical exam and imaging are required in rare cases of pediatric traumatic hip joint dislocation, with prompt closed reduction, in order to achieve favorable clinical outcomes.

## CRediT authorship contribution statement

**Mirza Sivro:** Formal analysis, Writing – review & editing, Conceptualization, Writing – original draft, Data curation. **Đemil Omerović:** Formal analysis, Supervision, Writing – review & editing, Conceptualization.

## Ethics statement

Ethical approval was obtained from the Ethics Committee of the Cantonal Hospital Zenica. Informed consent for the study and image publication was obtained from parents of the patient.

## Declaration of competing interest

The authors declare that they have no known competing financial interests or personal relationships that could have appeared to influence the work reported in this paper.

This research did not receive any specific grant from funding agencies in the public, commercial, or not-for-profit sectors.
